# Diagnostic importance of bone marrow aspiration evaluation: A single-center study

**DOI:** 10.12669/pjms.38.4.4797

**Published:** 2022

**Authors:** Ali Dogan, Sinan Demircioglu

**Affiliations:** 1 Ali Dogan, Van Yuzuncu Yil University, Faculty of Medicine, Hematology Department, Van, Turkey; 2 Sinan Demircioglu, Van Yuzuncu Yil University, Faculty of Medicine, Hematology Department, Van, Turkey

**Keywords:** Bone marrow aspiration, Bone marrow biopsy, Evaluation

## Abstract

**Background & Objective:**

Early diagnosis can be made based on the morphological examination of bone marrow aspiration (BMA) until the bone marrow biopsy (BMB) result is reported. This allows for treatment to be started immediately, especially in hematological malignancies for which urgent treatment is indicated. This study aimed to determine the sensitivity and importance of bone marrow aspiration in the diagnosis of hematological malignancies.

**Methods:**

In this study, the data of patients who underwent bone marrow aspiration and bone marrow biopsy in Van Yuzuncu Yil University hospital between 2017 and 2019 were retrospectively analyzed. A total of 500 patients who simultaneously underwent BMA and BMB were included in the study. Data were obtained from electronic medical records.

**Results:**

Indication for bone marrow evaluation was abnormalities in complete blood count in 270 (54%) of patients. The diagnosis was made based on the evaluation of BMA in 475 (95%). In 456 (96%) of the 475 patients diagnosed with BMA, the diagnosis was consistent with that of BMB. Agreement of BMB with BMA was 100% in acute and chronic leukemias, while BMA was not sufficient for the diagnosis of lymphoma and solid organ metastasis.

**Conclusion:**

Our study showed that the evaluation of BMA was highly sensitive in the diagnosis of hematological malignancies, such as acute leukemias, chronic leukemias, and multiple myeloma.

## INTRODUCTION

As a large organ mass, bone marrow is not only the source of many hematological diseases but also a site of involvement of some infections, solid organ metastases, and metabolic diseases.^1^ Effects on bone marrow manifest in abnormal laboratory values, lymphadenopathy, splenomegaly, and disease-specific clinical symptoms.^1^ The examination of bone marrow in many blood diseases is an important diagnostic tool following the assessment of medical history, physical examination, blood count analysis, and examination of peripheral smear morphology. In such cases, it is of great importance to examine using bone marrow aspiration and bone marrow biopsy procedures, which are usually, performed simultaneously.^2^

BMA is mainly performed for the cytomorphological examination of bone marrow cells. It also allows taking blood samples for other analysis such as flow cytometry, cytogenetics, molecular genetics, microbiological tests, and immunophenotyping.^3^ The morphological examination of BMA offers an accurate and early diagnosis of some hematological malignancies within a few hours until the result of BMB is reported within an average of 10-14 days. Thus, treatment of life-threatening malignancies that require urgent treatment, such as acute and chronic leukemias and multiple myeloma can be started immediately. In this study, we aimed to investigate the sensitivity of BMA for the diagnosis of hematological malignancies and evaluate the compatibility between BMA and BMB.

## METHODS

A total of 500 patients who underwent simultaneously BMA and BMB in Van Yuzuncu Yil University Medical Faculty Hospital between 2017 and 2019 were included in the study. Patients with and age ≥18 were included. The patient files and data recorded in the hospital’s information system were analyzed retrospectively. The bone marrow examination was performed from the posterior superior of the iliac crest and anterior superior of the iliac crest in patients. For BMA examination, 0.5 ml of bone marrow blood was aspirated at the first aspirate. In addition, bone marrow biopsy material of ≥1 centimeter was taken for BMB examination.

The demographic data, laboratory values of the patients, peripheral smear findings, bone marrow aspiration, and biopsy indications were examined. As a result of these preliminary evaluations, in patients with suspected hematological malignancies, blood samples for flow cytometry and genetic mutation analysis were taken from the area where BMA/BMB had been performed. BMB material sampled from the place where BMA had been performed or from a different area was transferred to the pathology laboratory in formol. The blood aspirated from bone marrow was spread on slides and stained with May-Grunwald-Giemsa staining, and then examined under a microscope at 10 and 100 magnifications. Cellularity and megakaryocytes were evaluated at 10 magnifications in the BMA examination. The morphology, maturation and differentiation characteristics of cells at 100 magnifications were determined by counting 400 cells and formulation. Subtyping of acute leukemias was made by evaluating flow cytometry. In cases where the examination of BMA was not sufficient, the definitive diagnosis was made based on the BMB result. The agreement between the diagnoses of BMA and BMB was assessed.

### Statistical Analysis:

Numerical data were expressed as mean ± standard deviation, and categorical variables as numbers and percentages (n, %). In the evaluation of BMA and BMB, if the diagnosis was the same, the result was accepted as consisted, and if the diagnoses of the two procedures differed, the result was considered as inconsistent. The rate of agreement between the two procedures was expressed as a percentage. Statistical analyses were performed using SPSS version 25.0 (IBM SPSS, Chicago, IL).

### Ethical approval:

Ethical approval for the study was obtained from the non-invasive clinical research ethics committee of Van Yuzuncu Yil University (approval date: 05.07.2019, decision number: 2019/11-03).

## RESULTS

Of the 500 patients included in the study, 274 (54.8%) were male and 226 (45.2%) were female, and the median age was 51.52±16.88 (18-87) years. A bone marrow examination was performed for diagnosis in 382 (76.4%) of the patients and for the evaluation of remission in 118 (23.6%). The most common indication for a bone marrow examination was pancytopenia (n = 83, 16.6%). The remaining indications are shown in Table-I. BMA was evaluated in 475 (95%) of the patients included in the sample, while it could not be evaluated in the remaining 25 (5%) patients due to insufficient particle sampling or poor staining. According to BMA evaluation, the most common diagnoses were hematological malignancies. Table-II presents the results of the BMA evaluations in all the patients. Among the 475 patients diagnosed based on the BMA evaluation, the diagnosis was consistent with the BMB report in 456 (96%) cases (Table-III). For a total of 44 (8.8%) cases, including 25 (5%) that could not be evaluated with BMA and 19 in which the BMB result was not consistent with the BMB report, the definitive diagnosis was made based on the BMB results, as shown in Table-IV.

**Table-I:** Bone marrow examination indications of the patients.

Indication	n (%)
***Laboratory findings***	
Pancytopenia	83 (16.6%)
Anemia + leukocytosis + thrombocytopenia	57 (11.4%)
Anemia	40 (8%)
Anemia + thrombocytopenia	23 (4.6%)
Albumin/globulin inversion + Ig increase	23 (4.6%)
Anemia + leukocytosis	17 (3.4%)
Leukocytosis	16 (3.2%)
Thrombocytopenia	11(2.2%)
Anemia + leukopenia	10 (2%)
Polycythemia	7 (1.4%)
Thrombocytopenia + leukopenia	6 (1.2%)
Thrombocytosis + leukocytosis	4 (0.8%)
***Clinical findings***	
Acute leukemia remission evaluation	69 (13.8%)
Lymphoma staging	41 (8.2%)
Multiple myeloma remission evaluation	37 (7.4%)
Splenomegaly	17 (3.4%)
Chronic leukemia remission evaluation	12 (2.4%)
Fever	4 (0.8%)
Other	23 (4.6%)

Ig: Immunoglobulin.

**Table-II:** Results of bone marrow aspiration.

Diagnosis	n (%)
Acute leukemia (AML + ALL)	99 (19.8%)
***Remission evaluation:***	
In remission	79 (15.8%)
Not in remission	30 (6%)
Normal bone marrow	74 (14.8%)
Multiple myeloma	44 (8.8%)
MDS	39 (7.8%)
Not evaluated	25 (5%)
CLL	22 (4.4%)
CML	21 (4.2%)
MPN other than CML	14 (2.8%)
Lymphoma infiltration	10 (2%)
Solid organ metastasis	9 (1.8%)
Hypocellular bone marrow	9 (1.8%)
Megaloblastic anemia	6 (1.2%)
Hairy cell leukemia	6 (1.2%)
ITP	6 (1.2%)
HLH	4 (0.8%)
Aplastic anemia	3 (0.6%)

AML: Acute myeloid leukemia; ALL: Acute lymphocytic leukemia; MDS: Myelodysplastic syndrome; CLL: Chronic lymphocytic leukemia; CML: Chronic myeloid leukemia; MPN: Myeloproliferative neoplasia: ITP: Immune thrombocytopenic purpura; HLH: Hemophagocytic lymphohistiocytosis.

**Table-III:** Diagnostic agreement between BMA and BMB.

BMA diagnosis	Agreement with BMB diagnosis
Acute leukemia (AML + ALL)	99/99 (100%)
***Remission evaluation:***	
In remission	78/79 (98.7%)
Not in remission	30/30 (100%)
Normal bone marrow	69/74 (93.2%)
Multiple myeloma	44/44 (100%)
MDS	33/39 (84.6%)
CLL	22/22 (100%)
CML	21/21 (100%)
MPN other than CML	11/14 (78.6%)
Lymphoma infiltration	10/10 (100%)
Solid organ metastasis	9/9 (100%)
Hypocellular bone marrow	5/9 (55.5%)
Megaloblastic anemia	6/6 (100%)
Hairy cell leukemia	6/6 (100%)
ITP (planned to undergo splenectomy)	6/6 (100%)
HLH	4/4 (100%)
Aplastic anemia	3/3 (100%)
Not evaluated	0/25 (0%)

BMA: Bone marrow aspiration; BMB: Bone marrow biopsy; AML: Acute myeloid leukemia, ALL: Acute lymphocytic leukemia; MDS: Myelodysplastic syndrome; CLL: Chronic lymphocytic leukemia; CML: Chronic myeloid leukemia; ITP: Immune thrombocytopenic purpura; MPN: Myeloproliferative neoplasia; HLH: Hemophagocytic lymphohistiocytosis.

**Table-IV:** The results of patients not diagnosed with BMA but diagnosed with BMB.

Diagnosis	44/500 (%8.8)
Normal bone marrow	15 (%3)
MDS	9 (%1.8)
Lymphoma infiltration	6 (%1.2)
Multiple myeloma	3 (%0.6)
Secondary metastasis	3 (%0.6)
Aplastic anemia	2 (%0.4)
MPN other than CML	2 (%0.4)
CMML	2 (%0.4)
LHH	1 (%0.2)
Megaloblastic anemia	1 (%0.2)

LHH: Hemophagocytic lymphohistiocytosis; CMML: Chronic myelomonocytic leukemia.

## DISCUSSION

Since acute and chronic leukemias show diffuse involvement in bone marrow, a BMA evaluation is usually sufficient when these malignancies are suspected.^2^ It is possible to differentiate Acute myeloid leukemia (AML) or Acute lymphocytic leukemia (ALL) according to the characteristics of blasts seen in the BMA. However, it may sometimes not be possible to distinguish acute leukemias by a morphological examination. Therefore, acute leukemias need to be subtyped for both treatment and follow-up purposes. In order to achieve this in the most accurate way, cells in blood samples taken by BMA should be evaluated with flow cytometry for immunophenotyping.^4^ In our study; flow cytometry was performed in all cases of acute and chronic leukemias. Flow cytometry is an effective method used in the diagnosis, differential diagnosis and follow-up of plasma cell neoplasms. In our study, we used flow cytometry in the differential diagnosis of multiple myeloma in patients with whom we considered plasma cell leukemia. In Eser A.’s study, the importance of flow cytometry, which is studied in the marrow blood sample taken with the first aspirate, is emphasized in the diagnosis and follow-up of multiple myeloma.^5^ It was easier to diagnose Chronic lymphocytic leukemia (CLL), Chronic myeloid leukemia (CML) and multiple myeloma with the evaluation of BMA due to the specific morphological appearance of bone marrow smear.

In a study by Bashawri, in which indications for a bone marrow examination and the most common diagnoses were determined in 1,813 patients presenting to a university hospital, the correlation between BMA and BMB diagnoses was evaluated. In that study, the most common indication for this examination was determined as the diagnosis and treatment of acute leukemia (n = 403, 22.2%), and BMA was concluded to be an important diagnostic tool in many conditions, especially in hematological diseases.^6^ In our study, the most common diagnosis based on BMA was acute leukemia (AML + ALL) detected in 99 (19.8%) patients, which was 100% consistent with the BMB report. In addition, in our study, there was 100% agreement between BMA and BMB diagnoses in patients with chronic leukemia such as CLL and CML. The retrospective analysis of the data of Calvet et al. revealed that BMA contributed to the diagnosis and treatment of 40 (20.7%) of 193 patients admitted to the intensive care unit. As a result, the authors concluded that BMA could significantly contribute to diagnosis and treatment in patients with or without any hematological malignancy during admission to the intensive care unit.^7^ In our study, the BMA evaluation directly contributed to the diagnosis and treatment of 200 (40%) patients, including 186 (37.2%) with malignant hematological diseases and 14 (2.8%) with benign hematological diseases. In a study by Gilotara et al. in 100 cases, when the results of BMA and BMB assessment were compared, there was a 72.4% agreement.^8^ Similarly, Toi et al. compared the results of simultaneously performed BMA and BMA in 160 cases and reported that the results of the two procedures were consistent for 61.25%.^9^ In the study of Puri et al., three hundred simultaneous BMA and BMB results were analyzed retrospectively. In this study, the overall agreement between BMA and BMB was 77.1%. The reason for the high level of general agreement between BMA and BMB in our study was the high adequacy ratio of BMB materials. In this study, the highest overall concordance rates between BMA and BMB were CLL, CML and acute leukemias, which were similar to the rates in our study.^10^ In our study, the diagnostic correlation between BMA and BMB was high at 91.2% among the 500 patients included in the sample. In hematological malignant diseases such as acute leukemia, chronic leukemia, and multiple myelomas, the success of BMA was even higher, and the diagnostic agreement between BMA and BMB was 100%.

In leukemias and other hematological malignancies with bone marrow involvement, in the interim period and after chemotherapy is completed, a re-examination of the bone marrow is required for the evaluation of remission.^11^ In our study, 79 patients were in remission according to BMA smear analysis. However, one of these patients was not in remission in BMB examination. In addition, 30 patients were found to be not in remission in both BMA and BMB examinations. In the study conducted by Puri et al., in the analysis of BMA and BMB results performed simultaneously in three hundred patients; In the evaluation of remission in patients with acute leukemia, CML and multiple myeloma, BMA and BMB were 100% compatible. Thus, in this study and in our study, BMA examination was found to be an effective diagnostic method in remission assessment.^10^

Although cell morphologies were adequately evaluated with BMA, in malignancies with focal involvement, such as lymphoma and multiple myeloma, the diagnosis is made based on BMB when there are not sufficient particles with aspiration or diagnostic cells.^12^,^13^ In our study, the results of BMA and BMB were fully consistent in the diagnosis of 44 (8.8%) patients with multiple myeloma. However, three (0.8%) patients with multiple myeloma could not be diagnosed with BMA due to focal involvement or insufficient particles, and the diagnosis was made using BMB. Similarly, for 10 (2%) patients diagnosed with lymphoma, lymphoma infiltration was present in both BMA and BMB, while three (0.6%) patients had lymphoma involvement only in BMB due to focal involvement or insufficient bone marrow in BMA.

In a study conducted by Phillips et al., bone marrow involvement was found in 759 (62.5%) patients in the bone marrow examination of a total of 1,215 patients diagnosed with lymphoma.**14** In our study, the bone marrow examination was performed for staging purposes in 41 patients with a definitive diagnosis of lymphoma, and was observed in 10 of these patients in the BMA evaluation, while an additional six patients were detected to have bone marrow involvement in BMB. Therefore, in our study, 16 (39%) of 41 patients who underwent lymphoma staging had bone marrow involvement and were considered stage-4. Metastasis of solid organ malignancies to the bone marrow is detected by the presence of non-hematological same type of cells in BMA examination.^15^ In our study, solid organ metastasis was detected in the BMA evaluation of nine (1.8%) cases. Similar to our study, Mansor et al. detected 2% solid organ metastases in the bone marrow examination of 1789 patients retrospectively.^16^

### Limitations of the study:

The design was retrospective and samples of a few cases could not be evaluated due to technical issues and problems about staining. However, our sample size is enough to determine the compatibilities between diagnosis via BMA and BMB.

## CONCLUSION

When the BMA evaluation is supported by flow cytometry and genetic analysis, almost all acute and chronic leukemias can be diagnosed. BMB is not necessary in the diagnosis of acute and chronic leukemia, except in cases where sufficient aspiration samples cannot be obtained for BMA evaluation. In the presence of the pre-diagnoses of lymphoma, multiple myeloma and solid organ metastasis, BMA should be performed together with BMB.

### Authors’ contributions:

**DA:** Was responsible for the accuracy and integrity of the study.

**DA, DS:** Analyzed and interpreted the data, prepared the manuscript, performed the statistical analyses, and were responsible for the final editing.
